# A Novel Scenario-Based, Mixed-Reality Platform for Training Nontechnical Skills of Battlefield First Aid: Prospective Interventional Study

**DOI:** 10.2196/40727

**Published:** 2022-12-06

**Authors:** Wenqiong Du, Xin Zhong, Yijun Jia, Renqing Jiang, Haoyang Yang, Zhao Ye, Zhaowen Zong

**Affiliations:** 1 State Key Laboratory of Trauma, Burn and Combined Injury Department for Combat Casualty Care Training, Training Base for Army Health Care Army Medical University Chongqing China

**Keywords:** mixed reality, decision-making, team work, battlefield first aid, nontechnical skills, training, next-generation modeling, virtual reality, medical education

## Abstract

**Background:**

Although battlefield first aid (BFA) training shares many common features with civilian training, such as the need to address technical skills and nontechnical skills (NTSs), it is more highly scenario-dependent. Studies into extended reality show clear benefits in medical training; however, the training effects of extended reality on NTSs, including teamwork and decision-making in BFA, have not been fully proven.

**Objective:**

The current study aimed to create and test a scenario-based, mixed-reality platform suitable for training NTSs in BFA.

**Methods:**

First, using next-generation modeling technology and an animation synchronization system, a 10-person offensive battle drill was established. Decision-making training software addressing basic principles of tactical combat casualty care was constructed and integrated into the scenarios with Unreal Engine 4 (Epic Games). Large-space teamwork and virtual interaction systems that made sense in the proposed platform were developed. Unreal Engine 4 and software engineering technology were used to combine modules to establish a mixed-reality BFA training platform. A total of 20 Grade 4 medical students were recruited to accept BFA training with the platform. Pretraining and posttraining tests were carried out in 2 forms to evaluate the training effectiveness: one was knowledge acquisition regarding the NTS and the other was a real-world, scenario-based test. In addition, the students were asked to rate their agreement with a series of survey items on a 5-point Likert scale.

**Results:**

A battlefield geographic environment, tactical scenarios, scenario-based decision software, large-space teamwork, and virtual interaction system modules were successfully developed and combined to establish the mixed-reality training platform for BFA. The posttraining score of the students’ knowledge acquisition was significantly higher than that of pretraining (t=−12.114; *P*≤.001). Furthermore, the NTS score and the total score that the students obtained in the real-world test were significantly higher than those before training (t=−17.756 and t=−21.354, respectively; *P*≤.001). However, there was no significant difference between the scores of technical skills that the students obtained before and after training. A posttraining survey revealed that the students found the platform helpful in improving NTSs for BFA, and they were confident in applying BFA skills after training. However, most trainees thought that the platform was not helpful for improving the technical skills of BFA, and 45% (9/20) of the trainees were not satisfied with the simulation effect.

**Conclusions:**

A scenario-based, mixed-reality platform was constructed in this study. In this platform, interaction of the movement of multiple players in a large space and the interaction of decision-making by the trainees between the real world and the virtual world were accomplished. The platform could improve the NTSs of BFA. Future works, including improvement of the simulation effects and development of a training platform that could effectively improve both the technical skills and NTSs of BFA, will be carried out.

## Introduction

Nontechnical skills (NTSs) are defined as “cognitive, social, and personal resource skills that complement technical skills, and contribute to safe and efficient task performance,” and decision-making and team cooperation are the 2 most important parts of NTSs in medicine [1,2]. Given that there is accumulating evidence for the positive impact of improved NTSs on patient outcomes [2], NTSs have gained much attention in medical training in recent years. Training for battlefield first aid (BFA) is a special branch of medical training. NTSs play an important role in BFA because the BFA providers face hostile fire when they rescue the injured. Decisions should be made and techniques should be chosen based on different tactical combat backgrounds [3,4]. In addition, the commander needs to synchronously organize combat and first aid, which requires close team cooperation compared with civilian first aid. Thus, NTSs should be addressed during BFA training.

There are currently several types of training methods for first aid, including lecture, practice on animal tissues, high-fidelity training simulators, and virtual reality (VR). Each method has its advantages and disadvantages [5]. Lectures are conducive to imparting knowledge, but not conducive to training first-aid skills. The use of live animal tissues, such as the chest wall or trachea covering from pigs, is suitable for training invasive first-aid skills such as needle decompression and cricothyrotomy; however, it is difficult to integrate the actual exercise into a tactical context [6]. High-fidelity mannequins can simulate various emergent conditions such as bleeding and airway obstruction, and they have been proven effective in improving trainees’ first-aid abilities. However, the limitations of these mannequins include their high cost, requirement of experienced operators, and poor integration into an actual tactical training environment [5,7]. In recent years, extended reality (XR), VR, augmented reality (AR), and mixed reality (MR), have been widely used in medical education, training, surgical planning, and the telementoring of complicated operations [8-10]. In addition, it has also been shown that VR, AR, and MR can improve the training effect of first-aid skills [11-13]. For example, Stone et al [13] developed an MR-based training platform for The UK Emergency Medical Team, which could blend the real-world objects of training relevance with VR reconstructions of operational contexts. Furthermore, AR has been shown to increase the accuracy and performance of medical students or medics in treating pneumothorax, airway management, and cardiopulmonary resuscitation training [8,11,12]. However, these XR-based methods have not been fully proven to effectively improve first-aid NTSs, especially the NTSs in BFA. The aim of this study was to create and test a tactical scenario-based, MR platform suitable for training the NTSs of BFA.

## Methods

### Ethical Considerations

All procedures were approved by the Ethics Committee of the Army Medical University of China PLA (approval #AMUWEC2020172) and were performed in accordance with the relevant regulations of the Ethics Committee of the Army Medical University of China PLA, China.

### Construction of a High-fidelity Platform for Training BFA

#### Establishment of Simulated Tactical Scenarios

To address first-aid skill training in tactical background and team cooperation in first aid, the training scenario was designed as a 10-person–based offensive battle drill conducted in mountainous terrain. One author with expertise in tactical command (ZY) was responsible for designing the training scenario. Briefly, the trained group selected a team leader. The trainees were then asked to siege a hill that was guarded by 3 soldiers. During the siege procedure, 2 trainees were programmed to be injured by hostile fire, and the team leader was asked to organize the hill-siege attack as well as the care of the injured personnel; the whole team was required to make correct decisions based on the tactical situation, cooperate, and complete the tactical task and BFA.

Based on the designed scenario, a 3D computer modeling of the scenario was established. First, high-precision models of soldiers, combat weapons, and armored vehicles were established by next-generation modeling technology. Second, animation of these objects and battle scenes were made based on the designed running variables and conditions. Third, the positions of the soldiers’ head in the real world were obtained by the simultaneous localization and mapping (SLAM) technique built into the VR glasses (head-mounted displays, HTC Vive Pro 2.0, High Technology Computer Corporation); the positions of the soldiers’ trunk in the real world were obtained by a home-made smart vest that contained location sensors (Chongqing High Tech Corporation); and the positions and movements of the simulated guns in the real world were captured by a built-in 9-axis sensor (Chongqing High Tech Corporation). These positions and movements were analyzed by an artificial intelligence system, and then an animation was generated in the virtual world. The actions of the 3 defending soldiers in the virtual world were trained by the artificial intelligence system in accordance with the script for action control. In this way, the interaction of the soldiers’ actions between the real world and the virtual world was accomplished.

#### 3D Modeling of the Simulated Battlefield Geographic Environment

As mentioned previously, the training scenario in this study was designed as an offensive battle taking place in a mountain area. Thus, several pictures of mountain areas were taken and used for the construction of the simulated battlefield geographic environment.

The 3D modeling of the geographical environment was divided into 5 layers: a terrain layer, surface layer, vegetation layer, building layer, and environment layer. The terrain layer was generated and restored by high-precision low-surface restoration technology and a 3D terrain-generation algorithm based on contour synthesis. On the basis of sampling and analyzing the surface material, the surface layer was constructed by composite material ball technology. The vegetation layer and the building layer were constructed by next-generation modeling technology in accordance with the simulated battlefield vegetation situation, training task, and imaginary enemy target situation. The environment layer simulated the real ambient light and weather corresponding to different combat times according to the training content.

### Construction of Software for Training of Decision-Making

To train the decision-making ability of the trainees, a decision tree was constructed. One author (ZZ), who had undergone tactical combat casualty care training in Ulm Hospital, Germany, was responsible for the construction of the decision tree. Key aspects of the basic principles of tactical combat casualty care, decision-making, injury state assessment, selection of appropriate first-aid tools, selection of appropriate first-aid skills, and important steps or details of each first-aid skill were listed. In total, 20 items were listed. Examples include “return fire first under hostile fire,” and “only apply tourniquet under hostile fire threat but no other procedures such as wound dressing for wounds with minor bleeding” [3,4]. The whole study group (the authors) consisted of the members of the Department of Training for Combat Casualty Care. There were 6 males and 1 female, with an average age of 34.6 (SD 5.28) years. We discussed and decided which items should ultimately be listed. Once the list had been completed, 1 question and 4 options were given for each item (Multimedia Appendix 1). The validity of the content was then checked by 6 trauma surgeons from Army Medical University based on questionnaires. These trauma surgeons were randomly selected from the Academic Committee on Trauma Care, Army Medical University. They were all men, with an average age of 52.3 (SD 6.47) years. These expert questionnaires were employed to evaluate the scientificity and feasibility of the testing standard. For the evaluation of scientificity, scores of 1, 3, 5, 7, and 9 indicated that the scientificity was very low, low, fair, high, and very high, respectively; for the evaluation of feasibility, scores of 1, 3, 5, 7, and 9 indicated that the testing standard was nonfeasible, fairly nonfeasible, feasible, fairly feasible, and highly feasible, respectively [14].

Unreal Engine 4 is a product of Epic Games, a top gaming company in the world [15,16]. It is one of the most widely used and sophisticated unreal engines at present. After continuous improvement, in addition to game development, it has also been used to perform film and television production, architectural design, car model building, urban planning, and factory assembly line simulation [16,17]. It provides a large number of core technologies needed by software developers, including the making of 3D game scenes, visual language, and C++ language. Blueprint is the visual language in Unreal Engine 4. It creates all kinds of executable processes in the form of “nodes” in advance, and then it can be easily programmed by the dragging of the mouse cursor to arrange and connect them. In this study, the constructed decision tree was visualized with the blueprint tool of Unreal Engine 4. An HTC Controller (HTC Vive Pro, High Technology Computer Corporation) was used by the participants to make choices in the virtual world.

### Construction of a Mobile, Large-Space, Team Collaboration and Virtual Interaction System

We used the SLAM skills [17,18] and spatial anchoring techniques to develop a large-space, multiplayer interaction between the real and the virtual worlds. Briefly, based on the virtual battlefield geographic environment established previously, a zero point was set at the center point in the virtual map, and the coordinates of the zero point were set as (0, 0, 0). According to the size of the training site, the coordinates of the training site in real world were then located. The real-world and virtual-world zero points were used for spatial anchoring.

During the drill, the starting coordinate was set as the birth point of the character in the virtual world. For example, if the birth position of the character’s virtual world was in the northeast coordinates (−250000, −250000, 0), then the real-world zero point was used for the spatial anchoring coordinates. When the character moved in the real world, the absolute motion coordinates of the virtual world could be obtained by the relative displacement between the dynamic coordinates and the spatial anchoring coordinates. The accurate dynamic coordinate relationship between the physical space and the virtual space was obtained by calculating the relative displacement through the spatial anchoring transformation. Based on the previous accurate coordinates of space anchoring, the world zeros (0, 0, 0) of multiple persons in the same physical real world were set at 1 point through the site calibration function of the positioning VR equipment, ensuring the consistency of the relative coordinates of multiple persons moving in the physical system and the relative position of the virtual world after anchoring. The relative motion of each person was transformed through the fixed anchor point and the virtual world patriarchal coordinate system, and the absolute position coordinates obtained were sent to each client in the form of a server network broadcast. After the client received the coordinates and unique information of different people, the picture was rendered through the 3D engine, and the reality was displayed in the VR glasses. In this way, the absolute coordinates of the virtual world could be compatible and synchronized in a large mobile space.

Once these separated modules had been constructed, Unreal Engine 4 and software engineering technology were used to combine these modules together to establish the high-fidelity training platform for BFA.

### Training Process and Testing Process

The study was designed as a randomized trial comparing the results of a pretraining test and a posttraining test. None of the recruited individuals had previously received any kind of training related to BFA. A total of 20 Grade 4 medical students from Army Medical University were recruited as trainees and were divided into 2 teams. There were 14 boys and 6 girls, with an average age of 20.6 (SD 1.28) years. The VR training was based on the basic principle of simulation-based training: an introduction and MR familiarization phase (30 minutes), a training phase (120 minutes), and then a debriefing phase (20 minutes) [19]. Exclusion and discontinuation criteria for the participants were the presence of VR sickness symptoms such as discomfort, headache, or nausea [19,20]. Ten rounds of training were conducted for each team to ensure that each of the students was able to experience different roles (team leader, team members responsible for fighting, team members responsible for first aid, and the simulated casualty).

Pretraining and posttraining tests were carried out in 2 forms: one form was the knowledge acquisition regarding the NTSs [19,21], and another form was a real-world, scenario-based test [5]. Scores of knowledge acquisition regarding the NTSs of BFA were collected [19,21]. One hour before training and one hour after training, the students were asked to take a knowledge acquisition test. The content of knowledge test was the same as the aforementioned decision-making test (Multimedia Appendix 1), and the difference between the pretraining and posttraining test was the scrambled order of questions and answers [21]. A higher score corresponded to a higher level of knowledge [19].

One day before the training and two hours after the training, the real-world, scenario-based test was performed [5]. In the real-world, scenario-based test, the scenario was similar to the tactical scenario used to establish the platform in the current study. The main difference was that 2 simulators (Trauma HAL S3040.100, Gaumard) were placed in the offensive route, serving as simulated injured personnel. The students were asked to fulfill the task and rescue the simulated casualties synchronously. The test standard for the real-world, scenario-based test was as previously reported but with small modifications [5]. The modified test standard consisted of 2 parts: NTSs (40 points) and technical skills (60 points). The total score of the test was 100 points (Multimedia Appendix 2). Total score and scores of each part were recorded and used for statistical evaluation.

### Posttest Survey

After the training, the trainees were asked to indicate their agreement with a series of survey items on a 5-point Likert scale (1=fully disagree, 2=disagree, 3=neutral, 4=agree, 5=fully agree) [22]. The research measures and questions are shown in Table 1.

**Table 1:** Measures and questions of posttraining survey.

Research measures	Questions
Confidence	1. I feel confident in applying my BFA^a^ skills quickly and correctly after training.
Helpfulness and simulation effect	2. I feel the simulation training approach was more efficient in improving technical first aid skills than the traditional approach. 3. The simulation effects of the battlefield geographic environment and battle scenario are very real. 4. I thought the platform was helpful in improving team cooperation. 5. The training platform was helpful in improving decision-making for first aid.

^a^BFA: battle-field first aid.

### Statistical Analysis

All data are expressed as the mean and SE. SPSS 11.0 (IBM Corporation) was used to analyze the results. *t* tests were used to compare the pretraining and posttraining scores. The CI was set at 95% (95% CI). A value of *P<*.05 was considered significant.

## Results

### The Constructed MR platform

**Figure 1:**
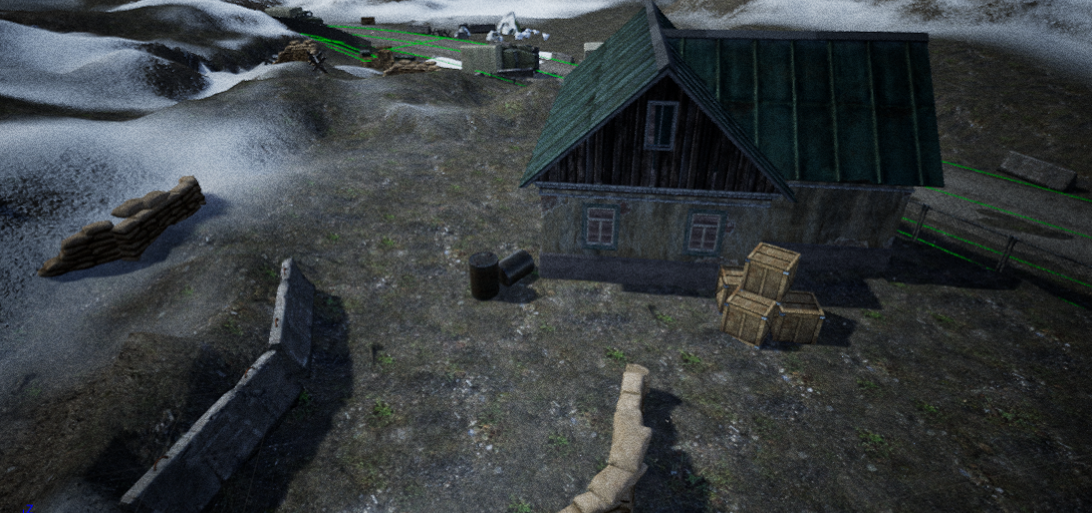
Exemplary picture of the constructed geographic environment.

**Figure 2:**
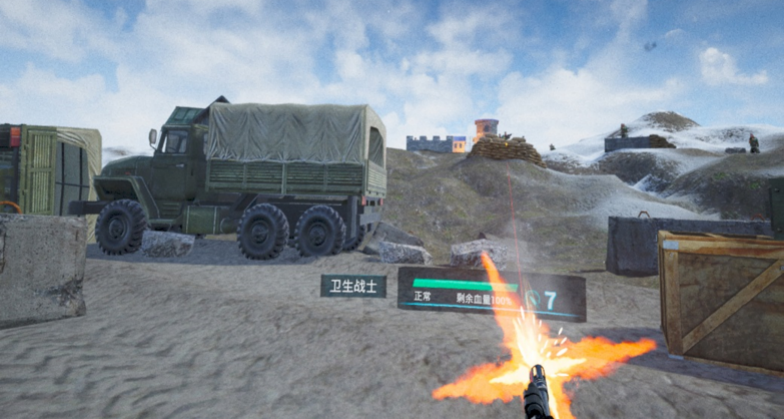
Exemplary picture of the constructed tactical scenarios.

Based on the designed scenario (ie, a 10-person–based offensive battle drill conducted in mountainous terrain), the battlefield geographic environment and tactical scenarios were successfully developed. Figure 1 is an exemplary picture of the constructed battlefield geographic environment and Figure 2 is an exemplary picture of the constructed tactical scenarios.

A scenario-based decision tree was established; dialog boxes were created focusing on the key issues of injury assessment, decision-making as to which first-aid equipment should be selected, and decision-making as to whether return fire should be performed first or casualty care should be performed first. The scientific score was 8.47 (SE 1.15), and the feasibility score was 8.26 (SE 1.13). These results revealed that the experts agreed with our determined decision-making questions. A total of 20 dialog boxes were constructed, covering the key components of the scenario-based first aid (Multimedia Appendix 1).

**Figure 3:**
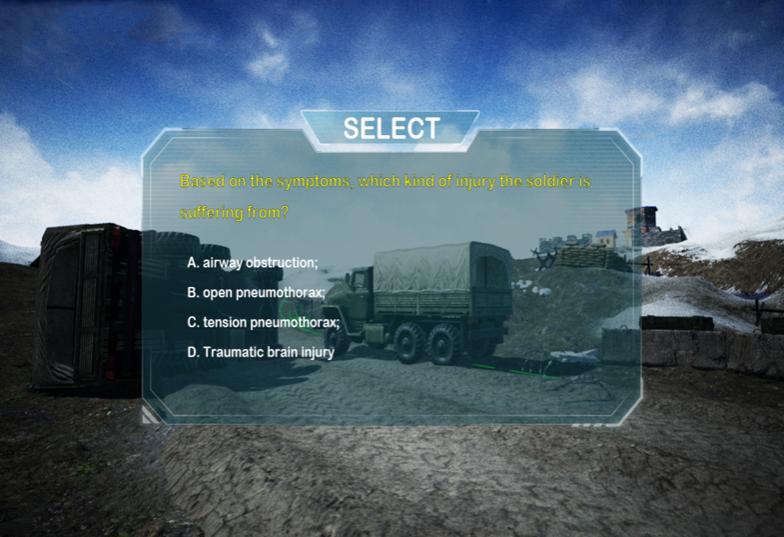
Screen capture of an exemplary dialog box for training to make a correct injury state assessment.

In each dialog box, several options were offered for each question, and the trainees were asked to select the right one(s) during training or testing. In the training model, if a wrong choice was made, the procedure returned to the previous step until the right answer was selected; in the test model, if a wrong choice was made, the procedure continued according to the choice. Figure 3 shows an exemplary dialogue box: when a soldier demonstrated shortness of breath, increased breath frequency (30-40/minute), and laborious breath due to facial injury, the trainees were asked to select one answer out of four options: (1) airway obstruction, (2) open pneumothorax, (3) tension pneumothorax, and (4) traumatic brain injury (Figure 3). In the training model, if a wrong choice was made, the procedure returned to the previous step until the right answer was selected. In contrast, in the test model, if a wrong choice was made, the procedure continued, but the corresponding points were deducted.

**Figure 4:**
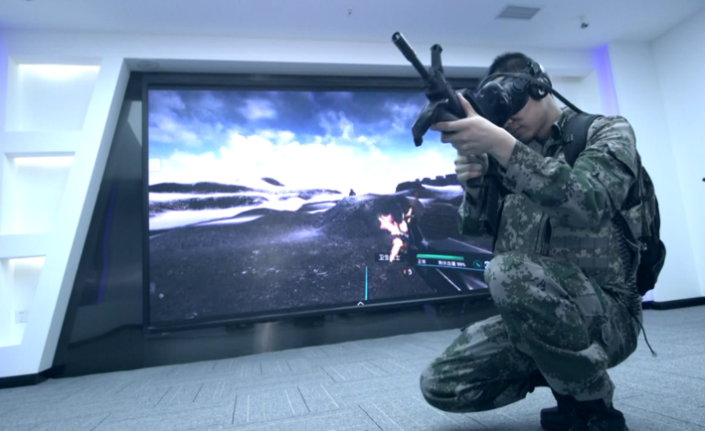
Exemplary picture showing the interaction between the real world and the virtual world.

A high-fidelity training platform for BFA was constructed by combining all the modules developed in the aforementioned step by Unreal Engine 4 and software engineering technology. The platform provided the whole-process experience of fighting against the enemy, taking casualties, conducting first aid for the casualties, and treating and completing the tactical tasks under the tactical background. By using spatial anchor sharing technology and an anchoring modification algorithm, the aims of team cooperation and MR (interaction between the visual world and the real world) were achieved. Figure 4 is an exemplary picture demonstrating the interaction between the real world and the virtual world.

### The Constructed Platform Was Helpful in Improving the NTSs of BFA

The students were divided into 2 teams to undergo training with the MR platform. During the training, the students moved freely, and they could cooperate with each other during the training. The leaders were in charge of the whole training process, making decisions and leading the teams to complete the mission (Multimedia Appendix 3). No student reported simulator-related sickness, such as discomfort, headache, or nausea.

The score that the students obtained in the knowledge acquisition test after the training was 17.35 (SE 1.35), which was significantly higher than that before training (t=−12.114; *P*≤.001). In the real-world, scenario-based test, the NTS score that the students obtained after the training was 30.5 (SE 2.67), which was significantly higher than that before training (22.1, SE 5.34; t=−17.756; *P*≤.001). In addition, the total score that the students obtained after the training was significantly higher than that before training (pretraining: 77.7, SE 3.17; posttraining: 66.45, SE 2.58; t=−21.354; *P*≤.001). However, there was no significant difference between the scores of technical skills that the students obtained before and after the training (*P*=.69). These data indicated that the constructed platform was helpful for improving the NTSs of BFA but was not helpful for improving the technical skills of BFA.

### Posttraining Survey

For question 1, students gave a mean score of 4.2, and most of the 20 students (n=18, 90%) felt confident in applying BFA skills quickly after training. For question 2, students gave a mean score of 2.3, and 65% (n=13) of the students disagreed and 30% (n=6) of the students were neutral regarding the following statement: “I feel the simulation training approach was more efficient in improving technical first-aid skills than the traditional approach.”

Moreover, 35% (7/20) of the participants disagreed and 40% (8/20) of the participants agreed with the following statement: “The simulation effect of the battlefield geographic environment and battle scenario is very real.” This indicated that the simulation effect of the constructed platform needed to be further improved.

For question 4, students gave a mean score of 4.4, and most students (18/20, 90%) thought the platform was helpful in improving team cooperation. For question 5, students gave a mean score of 4.75, and all of the participants (20/20, 100%) thought that the training platform was helpful in improving decision-making for first aid.

## Discussion

### Principal Findings

This study established a novel, MR platform that met the need for scenario-dependent decision-making and teamwork in the training field of combat first aid. First, a 10-person–based offensive battle taking place in a mountain area was designed as a tactical scenario; on that basis, simulated tactical scenarios and a battlefield geographic environment were established by next-generation modeling technology and an animation synchronization system. This enabled us to train first-aid skills in a tactical background. Second, decision-making training software addressing basic principles of tactical combat casualty care was constructed, making decision-making training possible. Third, a large-space team cooperation and virtual interaction system suitable for the simulation training system environment was developed, making teamwork training possible. Comparison of the performance of the pretraining and the posttraining tests showed that the platform constructed in this study was helpful in improving the NTSs of BFA. Furthermore, the posttest survey revealed that the students agreed that the platform was helpful in improving team cooperation and decision-making for first aid, and they were more confident in applying BFA skills after the training. However, most trainees thought that the platform was not helpful in improving technical skills in first aid, and 45% (9/20) of the trainees were not satisfied with the simulation effect of the battlefield geographic environment and battle scenario.

### Comparison to Prior Work

High-level NTSs are of paramount significance for safe and effective medical care, especially in first aid, where the time for making decisions and taking measures is short. In recent years, behavior marker systems, first-person perspective video, and high-fidelity simulators have been shown to be effective in improving the NTS performance of first aid [2,23,24]. To the best of our knowledge, however, there are no XR-based simulators specifically focusing on the training of NTSs for first aid, and the current study is the first to focus on the training of BFA NTSs. The existing XR-based simulators reported in the literature mainly focused on single-skill training or on making the trainees familiar with the environments, but NTSs including decision-making and teamwork of first aid have seldom been addressed [20,25]. In addition, training NTSs of first aid using XR-based simulators is considered technically difficult [13,19,26].

A multiplayer design within the XR environment is thought to be able to offer a chance to rotate through different team roles, supporting an active and immersive learning experience with the potential to equip the learners with the crucial teamwork skills required for medical care [25]. Chheang et al [27] established anesthesia simulation software and laparoscopic simulation software that were combined within a multiuser VR environment; the interaction of multiplayers could be fulfilled in the virtual world. It was revealed that the multiuser-based software could improve problem-based communication during surgery. Krishnappa et al [28] developed a multiplayer interaction platform using VR augmented with eye-tracking technology, making children with autism spectrum disorder practice gaze sharing and gaze following in a team possible [28]. In our study, SLAM skills along with spatial anchoring techniques were used to make the movement and cooperation among 10 people possible. In addition, the interaction between the real world and the virtual world was accomplished. To the best of our knowledge, this is the first paper of its kind in the field of first-aid training. The multiplayer experience and the interaction between the real world and the virtual world are helpful for the improvement of team work [2].

There is accumulating evidence showing the effectiveness of an XR-based simulator in improving medical skills; however, the underlying mechanisms for the effectiveness are controversial. Many factors, including the quality of the animation in XR, the additional physical stimuli, and visually induced motion sickness, are considered to affect the training effectiveness through attractiveness, motivation, or goal-oriented training procedures. Checa et al [29] found that immersive VR environments could enhance autonomous learning compared with a conventional lecture or the same serious game on a desktop computer and that novelty might play a role in the improved training effectiveness. In a study comparing the effects of tablet-based and VR-based serious gaming modules for basic life support training on learning outcomes, Aksoy et al [21] found that motivation by VR contributed to enhanced learning outcome [21]. In this study, 45% (9/20) of the trainees were not satisfied with the simulation effect of the battlefield geographic environment and battle scenario; however, the platform was able to effectively improve NTSs of BFA. Thus, we postulated that the improved NTSs might not be caused by the attractiveness of the simulated battlefield geographic environment and battle scenario; instead, we believe that the team work and decision-making–oriented design (ie, the decision tree incorporated in the software and the team cooperation design in the software) contributed to the improved NTSs.

### Strengths and Limitations

This study has several strengths. To our knowledge, this is the first MR-based platform for training NTSs in BFA, and the training effectiveness was satisfactory. In addition, the interaction of 10 persons in a large space between the real world and the virtual world was accomplished for the first time in the field of first-aid training.

This study also has some limitations. First, the simulation effect of the geographic environment and battle scenario need to be greatly improved. The posttraining survey revealed that the students were not highly satisfied with the simulation effect. This quality of the simulated battlefield geographic environment and battle scenario probably negatively affected the training effectiveness. The reason for the relatively low quality of simulation is related to the limited budget. Thus, in the future, efforts will be made to further improve the simulation effect when enough funding is available. Second, invasive first-aid techniques and technical first-aid skills could not be trained in the current platform, and this had a negative impact on the training effectiveness as confirmed by the finding that training with the platform did not improve the score of technical skills in the real-world test. AR has been shown to be beneficial for invasive first-aid techniques training; thus, the combination of techniques used in the current study with XR techniques might be a good option to address more challenges faced by BFA training.

### Future Directions

Given the strengths and limitations of this study, a training platform that could effectively improve both the technical skills and NTSs of BFA will be developed. In addition, efforts will be made to improve the simulation effect of the battlefield geographic environment and battle scenario; following this, the role of the improved quality of simulation in training effectiveness will be investigated with the current platform as a control.

### Conclusions

A novel scenario-based, MR platform was constructed in this study. In this platform, interaction of the movement of 10 people in a large space between the real world and the virtual world was accomplished with SLAM skills along with spatial anchoring techniques, and the interaction of decision-making by the trainee between the real world and the virtual world was fulfilled by the HTC Controller. The constructed platform could improve the NTSs (ie, team work and decision-making) of BFA. Future works, including improvement of the simulation effects and development a training platform that could effectively improve both the technical skills and NTSs of BFA, will be carried out.

## Appendix

Multimedia Appendix 1 The list of the 20 multiple-choice questions for assessing the knowledge of decision-making.

Multimedia Appendix 2 Testing standards used in the study.

Multimedia Appendix 3 Video demonstrating the training process.

## Abbreviations

AR: augmented reality
BFA: battlefield first aid
MR: mixed reality
NTS: nontechnical skill
SLAM: simultaneous localization and mapping
VR: virtual reality
XR: extended reality

## Data Availability

The data sets generated during and/or analyzed during the current study are available from the corresponding author (ZZ) on reasonable request.
