# Supplementary material for: A Novel Scenario-Based, Mixed-Reality Platform for Training Nontechnical Skills of Battlefield First Aid: Prospective Interventional Study
Source: JMIR Serious Games. 2022 Dec 6;10(4):e40727. doi: 10.2196/40727 (PMC9768658; doi:10.2196/40727)
Supplement: Multimedia Appendix 1 [file games_v10i4e40727_app1.doc]

Multimedia Appendix 1 The lists of 20 multiple-choice questions for assessing the knowledge of decision-making.

1. What are factors that influence decision making in battlefield first aid?
2. Hostile fire
3. Wounding patterns
4. Equipment constraints
5. Delays in reaching higher levels of care

Answer(s): A, B, C, D

1. During the phase of attacking, one of your team members is injured, and the injury state is current unclear. What should not be done?
2. If possible, deploy smoke upwind
3. If the wounded can help themselves, guide him to perform self-first aid
4. suppress enemy's fire
5. Ignore enemy fire and perform buddy first aid no matter

Answer(s): D

1. During the phase of attacking, 3 of your team members are injured and 2 of the injured can not move after injury. As a team leader, which of the following is the is the best choice?
2. Leave one person take care of the injured, and order others to fulfil the mission.
3. Ignore the injured and concentrate on the mission.
4. Leave 4 persons to take care of the injured, and order others to fulfil the mission.
5. Stop the mission. Rescue the injured and move them to safe place.

Answer(s): A

1. If you are one of the team members and you are unfortunate being shot on the left leg. You are unable to move and your team members are not available to help you now. Which of the following is the most inappropriate choice?
2. Lie down, keep still and pretend to be dead.
3. Use sign language for help.
4. Try to find and move to cover; apply tourniquet if situation permitted.
5. Howl loudly to attract the attention of your team members.

Answer(s): D

1. What is the most essential treatment task in Care Under Fire?
2. To treat the most immediate life-threatening injuries with tourniquet application on the battlefield.
3. Take cover, return fire, and gain fire superiority
4. Application of splint.
5. Keep the injured warm.

Answer(s): A

1. When performing first aid, which of the following’s first aid kit be preferred used?
2. Whatever.
3. Use the casualty’s first aid kit.
4. Use yourselves’.
5. Try to find substitute all the time.

Answer(s): B

1. What are the signs of life-threatening bleeding?
2. Bright red blood is pooling on the ground
3. The overlying clothes are soaked with blood
4. There is a traumatic AMPUTATION of an arm or leg
5. There is pulsatile (pulsing) or steady bleeding from the wound

Answer(s): A, B, C, D

1. Which of the following is the most suitable for hemorrhage control of major limb bleeding?
2. Tourniquet
3. Emergency Pressure Bandage
4. Triangle Bandage
5. Figure compression

Answer(s): A

1. Which of the following is the most suitable for moving the casualty in Care Under Fire?
2. one-person drag
3. two-person carry
4. Throne carry
5. None of the above

Answer(s): A

1. What is the proper distance a tourniquet should be placed in Care Under Fire?
2. 2 to 3 inches above the bleeding site
3. Tight and high
4. Whatever
5. 2 to 3 inches below the bleeding site

Answer(s): B

1. One of the team members was injured in the face, and the casualty demonstrated shortness of breath, increased breath frequency (30–40/minute), laborious breath, and making snoring or gurgling sounds. Which of the following best describe his most urgent state?
2. Airway obstruction.
3. Soft tissue injury of the face.
4. Tension pneumothorax.
5. Head injury.

Answer(s): A

1. What is the best position for a conscious casualty with maxillofacial trauma?
2. Recovery position
3. Sit up & forward position
4. Prone position
5. Whatever

Answer(s): B

1. What is the best position for an unconscious casualty?
2. Recovery position
3. Sit up & forward position
4. Prone position
5. Supine position

Answer(s): A

1. One of the team members was shot in the right back. He demonstrated distress with increased respiration rates after injury. Which kind of injury the soldier is most likely suffering from?
2. Open pneumothorax
3. Airway obstruction
4. Tension pneumothorax
5. Major peritoneal bleeding

Answer(s): C

1. One casualty demonstrated increased respiration rates and distress 30 minutes after application of chest seal for open pneumothorax. What of the following measure (S) should be done?
2. Doing nothing.
3. Massive fluid resuscitation.
4. Needle decompression; burp the seal.
5. Add packing on the chest wound.

Answer(s): C

1. What are the best TACTICAL indicators of shock in Care Under Fire?
2. Decreased state of consciousness (if casualty has not suffered a head injury) and/or an abnormal, weak, absent radial pulse.
3. Measured radial blood pressure lower than 90 mmHg.
4. Measured urine output less than 50 ml per hour.
5. Lactate≥mmol/L.

Answer(s): A

1. What kind of dressing should be used on penetrating eye trauma with an impaled object?
2. Pressure dressing with an Emergency Pressure Bandage.
3. Pressure dressing with a triangle bandage.
4. Apply rigid eye shield, followed by pressure dressing.
5. Cut a hole in eye shield to allow the object to fit through, and protect the injury eye with it. Then dressing the injured eye.

Answer(s): D

1. What should you do first when you encounter a casualty with a thermal burn in Care Under Fire?
2. Open the airway.
3. Fluid resuscitation.
4. Dressing the wound.
5. Stop the source of the burn

Answer(s): D

1. What should you assess before and after splinting?
2. Circulation – pulse check
3. Motor – movement
4. Sensory – feeling
5. Responsiveness.

Answer(s): D

1. As a first aider, with whom do you communicate in a casualty situation in Care Under Fire?
2. The casualty
3. The tactical leader
4. Medical personnel upon arrival
5. None

Answer(s): A,B,C
