# Supplementary material for: A Novel Scenario-Based, Mixed-Reality Platform for Training Nontechnical Skills of Battlefield First Aid: Prospective Interventional Study
Source: JMIR Serious Games. 2022 Dec 6;10(4):e40727. doi: 10.2196/40727 (PMC9768658; doi:10.2196/40727)
Supplement: Multimedia Appendix 2 [file games_v10i4e40727_app2.docx]

**Multimedia Appendix 2 Testing standards used in the current study**

| **Major Parts** | **Score Indicators and Score Standards** |
| --- | --- |
| **Non-technical skills**: team work and decision making (score: 40 points) | Ten team members had a clear division of labor and performed their respective duties (5 points). |
|  | Language communication between team members was smooth. If there were arguments, team members could make a quick discussion and reach an agreement (5 points). |
|  | Good combination of tactics with medicine, such as first aid should only be carried out after the wounded were transferred to a safe position (5 points). |
|  | Casualties during the attacking process should be less than 3 (5 points). For every killed team member over 3, 2 points will be deducted out of 5 points maximum. |
|  | Make a correct decision to the raised questions related to the decision-making (20 points). One point for each question. |
| **Technical skills**: Timely and proper first aids for life-threatening conditions (score: 60 points) | Recognize the injury state (10 points). |
|  | The whole procedure of tourniquet application and wound dressing is smooth. The tourniquet should be tied 5-10 cm near the heart end of the simulated wound (5 points). |
|  | Bleeding was stopped (5 points). |
|  | Time to complete tourniquet application and wound dressing was less than 3 minutes (5 points). For every 30 seconds over the required time, 1point will be deducted out of 5 points maximum. |
|  | Could open the airway by proper methods (5 points). |
|  | Thyrocricocentesis was performed at appropriate position and depth (5 points). |
|  | Put the wounded at recovery position correctly (5 points). |
|  | Time to complete airway management was less than 3 minutes (5 points). For every 30 seconds over the required time, 1point will be deducted out of 5 points maximum. |
|  | Needle was put at appropriate position and depth (5 points). |
|  | Symptoms of tension pneumothorax was relieved (5 points). |
|  | Time to complete needle decompression was less than 2 minutes (5points). For every 10 seconds over the required time, 1point will be deducted out of 5 points maximum. |
